# The Effect of Sulforaphane on Glyoxalase I Expression and Activity in Peripheral Blood Mononuclear Cells

**DOI:** 10.3390/nu10111773

**Published:** 2018-11-15

**Authors:** Michela Alfarano, Donato Pastore, Vincenzo Fogliano, Casper G. Schalkwijk, Teresa Oliviero

**Affiliations:** 1 Department of Agricultural, Food and Environmental Sciences, University of Foggia, Via Napoli 25, 71122 Foggia, Italy; michela.alfarano@unifg.it (M.A.); donato.pastore@unifg.it (D.P.); 2 Food Quality and Design Chair Group, Department of Agrotechnology and Food Sciences, Wageningen University, P.O. Box 17, 6700 AA Wageningen, The Netherlands; vincenzo.fogliano@wur.nl; 3 Department of Internal Medicine, Maastricht University Medical Centre and CARIM School for Cardiovascular Diseases, P.O. Box 5800, 6202 AZ Maastricht, The Netherlands; c.schalkwijk@maastrichtuniversity.nl

**Keywords:** glyoxalase 1, sulforaphane, peripheral blood mononuclear cells, glutathione, glutathione-S-transferase

## Abstract

Studies demonstrate that the potential health-beneficial effect of sulforaphane (SR), a compound formed in broccoli, is the result of a number of mechanisms including upregulation of phase two detoxification enzymes. Recent studies suggest that SR increases expression/activity of glyoxalase 1 (Glo1), an enzyme involved in the degradation of methylglyoxal, is major precursor of advanced glycation end products. Those compounds are associated with diabetes complications and other age-related diseases. In this study, the effect of SR on the expression/activity of Glo1 in peripheral blood mononuclear cells (PBMCs) from 8 healthy volunteers was investigated. PBMCs were isolated and incubated with SR (2.5 μM-concentration achievable by consuming a broccoli portion) for 24 h and 48 h. Glo1 activity/expression, reduced glutathione (GSH), and glutathione-S-transferase gene expression were measured. Glo1 activity was not affected while after 48 h a slight but significant increase of its gene expression (1.03-fold) was observed. GSTP1 expression slightly increased after 24 h incubation (1.08-fold) while the expressions of isoform GSTT2 and GSTM2 were below the limit of detection. GSH sharply decreased, suggesting the formation of GSH-SR adducts that may have an impact SR availability. Those results suggest that a regular exposure to SR by broccoli consumption or SR supplements may enhance Glo1.

## 1. Introduction

**Figure 1:**
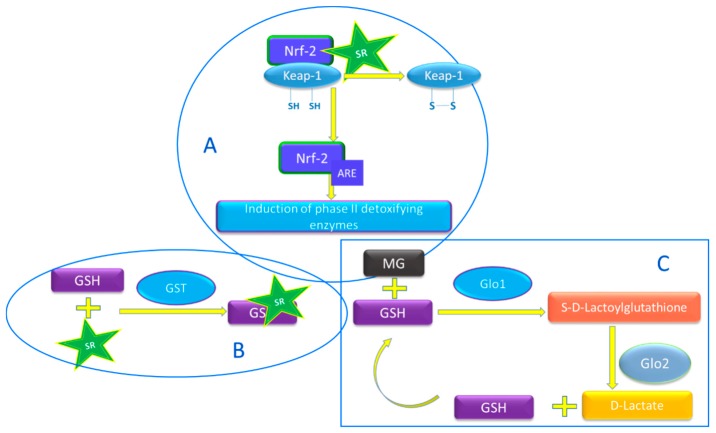
(**A**) Schematic representation of the potential effect of sulforaphane (SR) on phase II enzymes. SR can interact with cysteine residues of Kelch-like ECH-associated protein 1 (Keap1), causing dissociation of Keap1 from the transcription factor NF-E2-related factor 2 (Nrf2), allowing Nrf2 translocation to the nucleus and gene activation through the antioxidant response element (ARE) which are situated upstream of the promoter region of the genes of many antioxidant and phase II biotransformation enzymes, among those glyoxalase 1 (Glo1) and glutathione S-transferases (GST) [6,7]. (**B**) Schematic representation of the GST action [7,8]. It catalyzes the conjugation of GSH with xenobiotic compounds for detoxification, in this case SR. (**C**) Schematic representation of the glyoxalase system. Glyoxalase 1 (Glo1) catalyzes the formation of S-2-hydroxyacylglutathione from hemithioacetal which forms non-enzymatically from reduced glutathione (GSH) and α-oxoaldehyde methylglyoxal (MGO) [9,10]. Glyoxalase 2 (Glo2) catalyzes the hydrolysis of *S*-2-hydroxyacylglutathione to the corresponding aldonic acid, regenerating GSH that is consumed in the Glo1-catalysed reaction [9,10].

Epidemiological studies have shown correlations between dietary intake of *Brassica* vegetable and lower occurrence of cancer and various chronic diseases [1,2,3]. Part of such health-beneficial effects has been ascribed to breakdown products of glucosinolates, secondary plant metabolites present in *Brassica* vegetables, which represent a defense tool against herbivores [4]. Among glucosinolates breakdown products, sulforaphane (SR) is formed in large amounts in broccoli (*Brassica oleracea* L. var. *italica*) [5]. The potential, beneficial effect of SR is the results of several molecular mechanisms [1,2,3]. Evidence supports the hypothesis that SR exerts a modulating effect on phase II enzymes, by its interaction with Kelch-like ECH-associated protein 1 (Keap-1) (Figure 1A) [6,7]. SR can interact with cysteine residues of Keap1, causing dissociation of Keap1 from Nrf2, resulting in the translocation of Nrf2 to the nucleus and gene activation through the antioxidant response element (ARE). ARE is situated upstream of the promoter region of the genes of many antioxidant and phase II enzymes [6,7], including GST and Glo1.

GST catalyzes the conjugation of reduced glutathione (GSH) with xenobiotic compounds for detoxification (Figure 1B) [7,8]. Glo1 is the key enzyme in the defense against the dicarbonyl compound methylglyoxal (MGO) (Figure 1C) [9]. Methylglyoxal, which is mainly formed as a by-product of glycolysis, is the major precursor in the formation of advanced glycation end products (AGEs) (Figure 1C) [9,10]. Glo1 catalyzes the formation of *S*-2-hydroxyacylglutathione from hemithioacetal which forms non-enzymatically from GSH and MGO (Figure 1C) [9,10]. Subsequently, glyoxalase 2 catalyzes the hydrolysis of *S*-2-hydroxyacylglutathione to d-lactate, accompanied by regeneration of GSH.

MGO is associated with diabetes and other age-related diseases and MGO-derived AGEs accumulate in most sites of complications in diabetes (kidney, retina, and atherosclerotic plaques) [9,10]. Moreover, recent findings demonstrated that MGO-derived AGEs play a role in promoting neurodegenerative diseases [11]. An increased expression of Glo1 may therefore be considered as a promising strategy against the accumulation of MGO and this may be achieved by the intake of dietary bioactive compounds [12]. In a recent intervention study on humans, the consumption for 8 weeks of resveratrol and hesperidin together, (compounds present in grapes and citrus fruit, respectively), led to an increase of GloI activity by 27% [13]. However, the daily doses used in that study cannot be achieved by a regular diet since those compounds are found in low concentrations is those fruits. 

Recent studies showed that SR may be a promising inducer of Glo1 (Figure 1C). Both human hepatoma HepG2 cells and BJ fibroblasts SR (2 μM) increased Glo1 expression and Glo1 activity by 2–3-fold [14]. In another study, SH-SY5Y neuroblastoma cells were incubated SR (2.5 μM) and after 24 h and 48 h, the Glo1 activity increased significantly [15]. Most importantly, the concentrations of SR that were tested in these in vitro systems can be achieved in blood by consuming broccoli (e.g., 200 g steamed broccoli) [16,17]. The accumulation of MGO-derived AGEs is higher in subjects with abnormal glucose metabolism, but occurs also in healthy subjects and accelerates the functional decline that occurs with aging [18,19]. Therefore, diets rich in compounds that enhance the expression of Glo1, could help in reducing the accumulation of MGO-derived AGEs.

The aim of this study was to investigate the effect of SR on the Glo1 expression and activity in peripheral blood mononuclear cells (PBMCs). For this purpose, PBMCs were isolated from blood taken from human volunteers and incubated with SR to simulate a daily consumption of a broccoli portion. Glo1 expression and activity, total glutathione concentration, and the GSTP1, GSTT2 and GSTM2 expression, which encode phase II detoxification enzyme, GST, were measured.

## 2. Materials and Methods

### 2.1. Chemicals

Chemicals and solvents at analytical and HPLC-grade purity were purchased from Sigma-Aldrich Co. (St. Louis, MO, USA). WST-1 Cell Proliferation Assay was purchased from Roche Diagnostics (Mannheim, Germany). d-, l-Sulforaphane (SR) was obtained from Merck, Germany (574215). HT Glutathione Assay Kit was purchased from Trevigen, Inc. (Gaithersburg, MD, USA). Glyoxalase I human recombinant, expressed in *E. coli* (SRP6125, Sigma) Glutathione S-Transferase from equine liver (G6511, Sigma). Other chemicals are reported in the sections where their use is described.

### 2.2. PBMC Isolation

Peripheral blood samples were collected from 8 healthy adult donors (4 women and 4 men aged between 30 and 55 years). All subjects gave their informed consent for inclusion before they participated in the study. The study was conducted in accordance with the Declaration of Helsinki.

About 50 mL of blood from each donor was collected into Cell Preparation Tube (CPT, 362782, Sanabio, Schönebeck, Germany) and Peripheral Blood Mononuclear Cells (PBMCs) were isolated as following. After blood collection, the CPT tubes were gently inverted about 10 times and centrifuged at 1800× *g* for 20 min at room temperature. After centrifugation, plasma was removed and the white cloudy layer, containing PBMCs, was transferred into a 15 mL tube and washed twice with Phosphate Buffered Saline (PBS) by centrifugation at 300× *g* for 10 min. Cell pellet was dissolved in 1 mL of RPMI-1640 medium supplemented with 10% heat-inactivated FCS and 1% penicillin-streptomycin, and cells were counted by using Trypan blue staining. 

### 2.3. Cell Culture and Treatments

PBMCs were grown in RPMI-1640 in the absence (control) and in the presence of 2.5 μM SR, at 37 °C, in a humidified incubator containing 5% CO_2_ for 24 h and 48 h. 

### 2.4. Assessment of PBMC Viability

Cell viability of PBMCs incubated in the absence (positive control) and in the presence of 2.5 μM SR for 24 h and 48 h was evaluated by using the WST-1 Cell Proliferation Assay (Roche). It is a colorimetric assay based on the cleavage of a tetrazolium salt, 4-[3-(4-Iodophenyl)-2-(4-nitrophenyl)-2*H*-5-tetrazolio]-1,3-benzene disulfonate (WST-1), by mitochondrial dehydrogenases in viable cells to form formazan, an orange compound which absorbs at the wavelength of 450 nm. Briefly, at the end of each experiment 10%, Triton X-100 was added to the culture medium of PBMCs used as negative control. After 15 min of incubation at 37 °C, cells were centrifuged at 219× *g* for 5 min and about 170 μL of supernatant was removed. At this point, 70 μL of fresh medium and 10 μL of WST-1 reagent were added and cells were incubated at 37 °C and 5% CO_2_ in a humidified atmosphere. The absorbance was measured at 450 nm every 30 min using a Multiskan Ascent reader (Thermo Labsystem) until OD of the positive control was around 1–1.5 value. The viability of both SR-treated cells and negative control was calculated as (%) variation in absorbance at 450 nm in comparison with the positive control. For each participant and incubation time, two measurements were performed. In the statistical test the averages of those duplicates were compared, thus 8 values per test were entered.

### 2.5. Glo1 Activity Assay

Glo1 activity in PBMC lysate was assessed according to the method initially described by McLelland and Thornalley (1990) [20] and recently adapted by Arai et al. (2014) [21], with minor modifications. Briefly, PBMCs were collected, washed once with PBS and lysed in cold 10 mM sodium phosphate buffer pH 7.0 supplemented with 0.1% Triton X-100 and inhibitor protease cocktail. The Glo1 activity assay was performed by the addition of increasing amounts (from 20 to 200 μL) of cell lysate to a reaction mixture (final volume = 1 mL) containing 50 mM sodium phosphate buffer at pH 6.6 and 3 mM MG/GSH mixture, which had been equilibrated at 37 °C for 10 min before sample addition. The reaction was monitored spectrophotometrically by following the absorbance increase at the wavelength of 240 nm due to the formation of *S*-d-lactoylglutathione (Cary 60 UV-Vis, Agilent Technologies, Santa Clara, CA, USA). The reaction rate, expressed as ΔA240/min, was calculated as the tangent of the experimental curve at which the highest variation of absorbance per min was reached. Enzymatic activity, expressed in U.E./mg of protein, was calculated using a molar absorption coefficient (Δμ240) equal to 2.86 mM^−1^cm^−1^. For each sample, four replicates were performed (replicates of the measurements). The protein content in PBMC lysate was determined by Bradford’s method using BSA as a standard (three replicates of the measurements for each sample). In the statistical test the averages of those triplicates were compared, thus a total of 8 values per participant and per incubation time were compared in the statistical test. 

### 2.6. Determination of the Reduced Glutathione (GSH) Content in the PBMC Lysate

The GSH content in PBMC lysate was determined spectrophotometrically by using HT Glutathione Assay Kit (Trevigen, Gaithersburg, MD, USA). In this assay, sulfhydryl groups of GSH reacts with 5,5′-dithiobis-2-nitrobenzoic acid (DTNB) to produce both a yellow colored 5-thio-2-nitrobenzoic acid (TNB), that absorbs at 405 nm or 414 nm, and the mixed disulfide, GSTNB, that is reduced by glutathione reductase to recycle the GSH and produce more TNB. The rate of TNB production is proportional to the concentration of detected glutathione in the sample. This method allows to measure the total glutathione because the oxidized glutathione (GSSG) present in the sample is reduced by glutathione reductase, present in the reaction mixture, to two molecules of GSH. To measure GSSG content, free thiols present in the reaction must be masked with 2 M of 4-Vinylpyridine. The concentration of GSH is calculated by subtracting the GSSG levels from the total (GSH plus GSSG) glutathione value.

After incubation PBMCs were collected, washed once with PBS, deproteinized with 5% (*w*/*v*) metaphosphoric acid and lysed by the addition of 0.1% Triton X-100 and inhibitor protease cocktail to the cell suspension. A Multiskan Ascent reader (Thermo Labsystem) and 96-well plates were used for measurements. The reaction medium (final volume of each well: 0.2 mL) consisted of 1X assay buffers and samples (untreated PBMC lysates or 4-Vinylpyridine-treated PBMC lysates for the determination of total glutathione and GSSG, respectively). The reaction was started by adding 150 μL of freshly-prepared reaction mix, containing DTNB and the glutathione reductase, and monitored by recording the absorbance increase at 414 nm, due to TNB production, at 1 min intervals over a 10 min period. The samples were analyzed in quadruplicate (replicates of the measurements), and the slope from the maximum linearity portion of each curve was determined. Glutathione (total and GSSG) concentrations for each sample, were determined by comparing the slope of the samples with that of the standard curve obtained by using a GSSG standard. GSH content was thus calculated and expressed in nmol/mg of protein. The protein content in PBMC lysate was determined by Bradford’s method using BSA as a standard as described for the Glo1 activity. In the statistical test the averages of those replicates were compared, thus a total of 8 values per participant and per incubation time were compared in the statistical test. 

### 2.7. Determination of Change in GSH Concentration after 24 h of Incubation with SR

The change in the GSH content of a standard solution prepared with the commercial GSH (Sigma Aldrich, Saint Louis, MO, USA) after 24 h of incubation with SR at 37 °C was evaluated according to Browne and Armstrong (1998), with slight modifications. A CLARIOstar microplate reader (BMG Labtech, Ortenberg, Germany) and 96-well plates were used for measurements. The assay was conducted in a mixture containing 50 mM sodium phosphate buffer (pH 8.0) plus 5 mM ethylenediaminetetraacetic acid (EDTA) and GSH in the absence (control), and in the presence of SR (2.5 and 50 μM). The same solutions were prepared with the addition of GST enzyme (1 U/mL). After the addition of 0.38 mM of o-phthaldialdehyde (1 mg/mL in ethanol), the reaction mixture was incubated at 37 °C for 15 min. Fluorescence was measured by using wavelengths of excitation and emission of 350 nm and 420 nm, respectively. The change in GSH concentration after SR treatment was calculated as (%) fluorescence decrease with respect to control. For each GSH concentration, three replicates were performed and those values were statistically analyzed.

### 2.8. Real Time PCR

After treatment, PBMCs were washed once with PBS before addition of Trizol reagent (750 μL/1 × 10^6^ cells) to lyse the cells, then cells were subsequently frozen. After thawing, samples were kept at room temperature for 5 min to ensure complete dissociation of nucleoprotein complexes. Next, chloroform (150 µL; Merck) was added to the PBMCs before shaking the samples vigorously for 15 s. After 15 min incubation (room temperature), the resulting mixture was centrifuged at 12,000× *g* for 15 min at 4 °C. The aqueous phase was transferred to a fresh tube containing 5 µL Glycogen (5 mg/mL (Fisher Scientific Emergo, Landsmeer, The Netherlands). Next, 375 µL 2-propanol (Sigma) was added and the samples were mixed by vortexing. After 10 min incubation (room temperature), the samples were centrifuged at 12,000× *g* for 10 min at 4 °C. Supernatant was removed and the RNA pellet was washed by adding 1 mL of 75% ethanol. Samples were vortexed and centrifuged at 12,000× *g* for 5 min at 4 °C. The washing step was repeated once. The RNA pellets were air dried for 20 min after complete removal of ethanol. The RNA pellet was dissolved in 20 µL RNAse free water. RNA (250 ng) was used to make cDNA using the iScript cDNA synthesis kit (Biorad 1708891), according to the manufacturer’s instructions, and for the rtPCR IQ SensiMix SYBR master mix (Bioline, London, UK). Next, cDNA (10 ng) was used for each Real Time PCR reaction on a Biorad CFX. The housekeeping genes were RPS18 and RPLPO. The list of primers used for the PCR are shown in Appendix A (Table A1). Each sample was analyzed one time, thus a total of 8 values per participant and per incubation time were compared in the statistical test. 

### 2.9. Statistical Analysis

A paired T-test has been used to determine significant difference for cell viability, Glo1 activity, Glo1, GSTP1 expression, and GSH concentration between the control cells and the cells incubated with SR (two tests, one to compare control and incubation at 24 h, and one test to compare control and 48 h incubation).

## 3. Results

### 3.1. Viability Test

**Figure 2:**
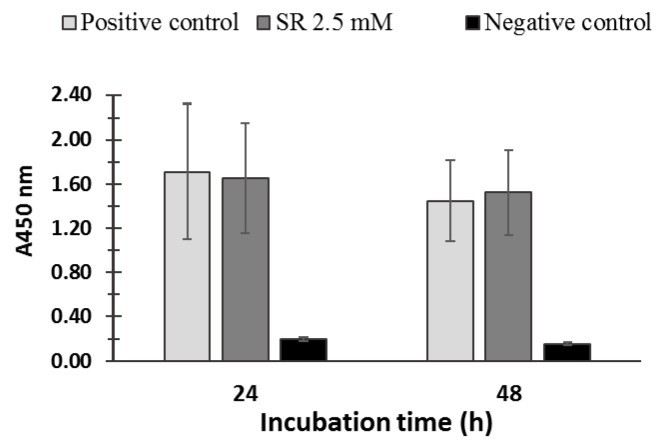
Cell viability of PBMCs incubated in the absence (positive control) and in the presence of 2.5 µM SR for 24 h and 48 h was evaluated by using the WST-1 Cell Proliferation Assay (Roche, Basel, Switzerland). The negative control represents the cells treated with Triton X-100. No significant differences were found between the positive controls and the SR-incubated cells (for both 24 h and 48 h incubations *p* > 0.05).

PBMCs viability was not significantly affected by SR (2.5 μM) treatment indicating no cytotoxic effect of SR on PBMC (Figure 2). Moreover, the potential cytotoxicity of dimethyl sulfoxide, the solvent in which SR was dissolved, was tested on PBMCs, and no cytotoxic effect was observed (data not shown).

### 3.2. Glo1 Expression and Activity

**Figure 3:**
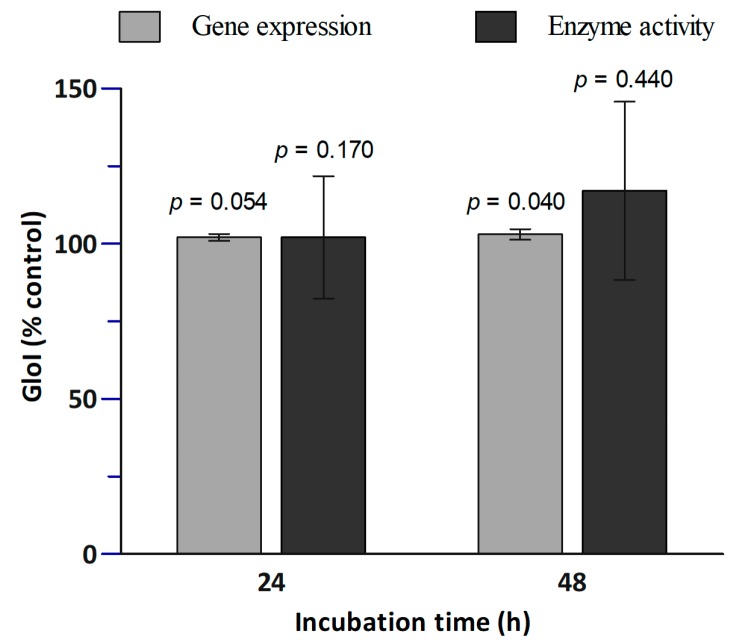
Effect of SR treatment on Glo1 gene expression and activity. PBMCs were treated with 2.5 µM SR for 24 h and 48 h followed by RT-PCR on Glo1 gene expression and Glo1 enzymatic activity assay. Data are shown as percentage of control: The bars at 24 h represent the expression and activity relative to the control at 24 h without SR, and the bars at 48 h represent the expression and activity relative to the control at 48 h without SR. Each bar represents mean ± SE; *n* = 8 subjects. The *p*-values were calculated by comparing the control values with 24 h SR incubation samples, and with the 48 h SR incubation samples.

In Figure 3, the effects of SR on both Glo1 gene expression and activity in PBMCs is shown. The bars at 24 h and at 48 h represent the expression and activity relative to the controls without SR (at 24 h and 48 h, respectively). SR did not significantly affect the Glo1 activity. However, after 48 h of SR treatment a slight significant increase (1.03-fold) of the gene expression was observed. 

### 3.3. GSH Levels

GSH plays an important role in the glyoxalase system, thus the effect of SR treatment on intracellular GSH levels was evaluated (Table 1).

**Figure 4:**
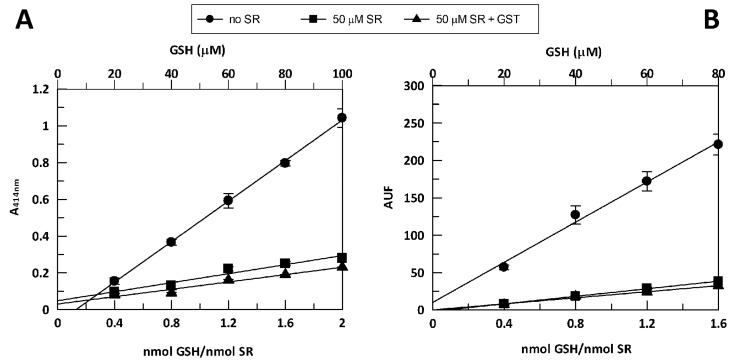
Effect of the presence of SR on GSH assayed in vitro. A GSH standard solution (20–100 µM in (**A**), 20–80 µM in (**B**)) was incubated for 24 h at 37 °C in the absence of SR (control) and in the presence of 50 µM SR alone or 50 µM SR + 1 U/mL GST. GSH levels were measured using both HT Glutathione Assay Kit (**A**) and the fluorescence probe *o*-phthaldialdehyde (**B**) as reported in Methods. Vertical bars represent ± S.D. (*n* = 3).

SR treatment caused a decrease in GSH levels compared to GSH levels in the control cells (Table 1), with a reduction by 73% and 61% of intracellular GSH concentration after 24 h and 48 h of SR treatment, respectively. To verify if the GSH concentration was reduced by the formation of adducts with SR, the direct chemical interaction between GSH and SR under our experimental conditions was tested (Figure 4). Different concentrations of GSH standard solution were incubated at 37 °C for 24 h in the absence of SR (control) and in the presence of SR (50 μM). GSH and SR concentrations were chosen to mimic the ratio GSH/SR achieved in the cell culture experiments. GSH levels were measured using two different methods. Data obtained from the two methods are plotted as A414 nm (2A) and Arbitrary Units of Fluorescence (AUF, 2B) vs. the ratio nmol GSH/nmol SR as well as vs. GSH concentration. In both cases, the presence of SR led to a reduction of the slope of the linear regression, hence to a reduction of free GSH. This suggests an interaction between GSH and SR and a possible formation of GSH-SR adducts that may have reduced the levels of GSH and SR in PBMCs. To understand if GST enzyme activity could have increased such adducts formation, GSH was also incubated at 37 °C for 24 h with 50 µM SR in the presence of 1 U/mL of GST (Figure 4). The linear regressions in presence (50 µM SR + GST) and in the absence of GST (50 µM SR) were similar, suggesting that the chemical conjugation between the two compounds was dominant.

### 3.4. GST Gene Expression

GSTT2 and GSTM2 expressions were too low to be detected while the values of GSTP1 are reported in Table 1. After 24 h, a slight but a significant increase of GSTP1 expression was measured (1.08-fold), while the expression was reduced after 48 h showing an insignificant difference with the control incubation.

## 4. Discussion

The design of the present study has been inspired by a few recent studies that have shown the potential of products naturally present in foods like SR in enhancing Glo1 expression and activity. PBMCs were chosen to obtain more insights on the effect of SR in vivo on healthy subjects. Therefore, one of the primary aims of this study was to investigate the effect of SR on the Glo1 expression and activity in PBMCs by incubating the cells in a concentration that is realistically achievable by consuming broccoli on a daily basis. In this study, by using such concentration, the cell viability did not change upon 24 h and 48 h incubation (Figure 2). Our result confirmed the results reported by Angeloni et al. (2015) [15] in which the same SR concentration as used in the present study did not affect the viability upon 24 h incubation of neuroblastoma cells. It is important to notice that in another study higher SR concentrations (10–40 µM) significantly reduced viability in human malignant mesothelioma cell line (MSTO-211H cells), at a concentration of 20 µM SR, and a significant reduction was also measured at a concentration of 10 µM after 72 h incubation [22]. In another study, in which 30 µM SR concentration was tested, a significant decrease in cell viability was observed after 72 h incubation in human breast cancer cell lines (MCF-7 and MDA-MB-231) [23].

In our study, the incubation of PBMCs from healthy volunteers with SR at physiological conditions, although at relatively low concentrations, led to a slight but significant increase of Glo1 expression (1.03-fold) (Figure 3). 

An increase of Glo1 expression and activity was measured in SH-SY5Y neuroblastoma cells treated with SR at the same concentration (2.5 µM) and at the same times (24 h and 48 h) [15]. In human hepatoma HepG2 cells and BJ fibroblasts the incubation with SR led to a significant increase of Glo1 activity and to a related dose-dependent increase in Glo1 mRNA levels [14]. Furthermore, in primary neonatal rat cardiomyocytes treated with SR (5 µM) for 24 h and subsequently exposed for other 24 h to MGO (1 mM), a significant lower MGO-induced damage was found [24]. 

Since Glo1 catalyzes the formation of *S*-d-lactoylglutathione from MGO and GSH, the intracellular concentration of GSH was measured to monitor the reaction [15,22,23]. Results showed that SR treatment caused 73% and 61% reduction in GSH levels compared to the control cells after 24 h and 48 h of SR treatment, respectively (Table 1). In contrast, in neuroblastoma cells (SH-SY5Y), SR treatment (24 h incubation) led to higher levels of GSH concentration [15,25]. We hypothesized that SR formed adducts with GSH led to upregulation of the expression of genes encoding GST. Isothiocyanates, such as SR, undergo reversible conjugation with GSH [8]. The data showed in Figure 4 indicates that the interaction between GSH and SR can lead to the formation of GSH-SR adducts. This is relevant because it may imply that less SR was available at the cellular level (Figure 3). The conjugation of SR with GSH was also observed in a study in which SR and GSH were incubated in sodium phosphate buffer (pH 6.5), where the addition of cloned human GST significantly increased the conjugation between GSH and SR [26]. However, in the present study, the addition of GST did not increase the conjugation between GSH and SR (Figure 3), suggesting that under the applied conditions, the chemical conjugation between the two compounds may have been prevalent. Moreover, we found a slight but significant increase of the expression of GSTP1 after 24 h incubation (1.08-fold), which was not observed after 48 h incubation. SR (0.1 μM), after 24 h incubation, led to an increase in GST activity (by 1.47-fold) in cells from hypertensive rats and a slight increase of the GST activity in normotensive cells from Wistar-Kyoto rats (at 0.5 µM SR) [27]. In a study in which human prostate cells (LNCaP) were incubated with 0.25–5 µM for 72 h, a dose dependent (5–10 µM) increase in GSH levels was observed after 48 h incubation and no effect was detected at doses comparable to those used in the present study [28]. Moreover, no changes in GSTA1 band (transcriptional response) appeared even after 72 h incubation [28].

## 5. Conclusions

In this study, the effect of SR on the expression/activity of Glo1 in PBMCs from healthy volunteers was investigated. PBMCs were isolated and incubated with an SR dose achievable by consuming a broccoli portion. Glo1 activity was not affected while a slight but significant increase of its gene expression (1.03-fold) was measured after 48 h incubation. Those results suggest that a regular exposure to SR from consumption of broccoli or SR supplements may enhance Glo1, consequently reducing the accumulation of MGO-derived AGEs.

Future research should investigate the effect of regular long-term administration of SR in vivo, as dietary broccoli or supplements, in health as well as pre-diabetic patients (in overweight patents with high blood glucose), on the Glo1 expression/activity and on MGO blood levels to establish the concentration and the frequency of SR intake able to significantly reduce MGO blood levels.

## Figures and Tables

**Table 1:** GSH content and GSTP1 gene expressions of PBMCs grown for 24 h and 48 h in the absence (control) and in the presence of SR (2.5 μM). Data are reported as absolute values (mean values ± S.E., *n* = 8 subjects). The *p*-values were calculated by comparing the control values with the 24 h SR incubation samples, and with the 48 h SR incubation samples.

Assay	24 h			48 h		
	Control	2.5 µM SR	*p*	Control	2.5 µM SR	*p*
GSH	34.75 ± 3.63 ^a^	9.49 ± 1.51 ^a^	<0.001	30.8 ± 3.26 ^a^	12.15 ± 1.35 ^a^	<0.001
GSTP1 gene expression	7.56 ± 0.27 ^b^	8.21 ± 0.17 ^b^	0.002	7.67 ± 0.17 ^b^	7.56 ± 0.32 ^b^	0.322

^a^ nmol/mg of protein; ^b^ ∆ CT.

## Appendix A

**Table A1:** Primers used for PCR.

RPS18	Ribosomal protein S18	Forward	ATCCTCAGTGAGTTCTCCCG
RPS18	Ribosomal protein S18	Reverse	CTTTGCCATCACTGCCATTA
RPLPO	Ribosomal protein P0	Forward	GCAATGTTGCCAGTGTCTG
RPLPO	Ribosomal protein P0	Reverse	GCCTTGACCTTTTCAGCAA
GLO1	Glyoxalase 1	Forward	GGTTTGAAGAACTGGGAGTCAAA
GLO1	Glyoxalase 1	Reverse	ATCCAGTAGCCATCAGGATCTTG
GSTP1	Glutation S-transferase 1	Forward	CTGCGCATGCTGCTGGCAGATC
GSTP1	Glutation S-transferase 1	Reverse	TTGGACTGGTACAGGGTGAGGTC
